# Emergence of dengue virus 4 genotype II in Guangzhou, China, 2010: Survey and molecular epidemiology of one community outbreak

**DOI:** 10.1186/1471-2334-12-87

**Published:** 2012-04-12

**Authors:** Qin-Long Jing, Zhi-Cong Yang, Lei Luo, Xin-Cai Xiao, Biao Di, Peng He, Chuan-Xi Fu, Ming Wang, Jia-Hai Lu

**Affiliations:** 1 Guangzhou Center for Disease Control and Prevention, Guangzhou 510440, People's Republic of China; 2 School of Public Health, Sun Yat-Sen University, Guangzhou 510080, People's Republic of China; 3 Zhongshan School of Medicine, Sun Yat-Sen University, Guangzhou 510080, People's Republic of China; 4 Key Laboratory of Tropical Disease Control, Sun Yat-sen University, Ministry of Education, Guangzhou 510080, People's Republic of China

## Abstract

**Background:**

The re-emergence of dengue virus 4 (DENV-4) has become a public health concern in South America, Southeast Asia and South Asia. However, it has not been known to have caused a local outbreak in China for the past 20 years. The purpose of this study was to elucidate the epidemiology of one local community outbreak caused by DENV-4 in Guangzhou city, China, in 2010; and to determine the molecular characteristics of the genotype II virus involved.

**Case presentations:**

During September and October of 2010, one imported case, a Guangzhou resident who travelled back from Thailand, resulted in 18 secondary autochthonous cases in Guangzhou City, with an incidence rate of 5.53 per 10,000 residents. In indigenous cases, 14 serum samples tested positive for IgM against DENV and 7 for IgG from a total of 15 submitted serum samples, accompanied by 5 DENV-4 isolates. With identical envelope gene nucleotide sequences, the two isolates (D10168-GZ from the imported index case and Guangzhou 10660 from the first isolate in the autochthonous cases) were grouped into DENV-4 genotype II after comparison to 32 previous DENV-4 isolates from GenBank that originated from different areas.

**Conclusions:**

Based on epidemiological and phylogenetic analyses, the outbreak, which was absent for 20 years after the DENV-4 genotype I outbreak in 1990, was confirmed as DENV-4 genotype II and initially traced to the imported index case, a Guangzhou resident who travelled back from Thailand.

## Background

Dengue is the most globally prevalent arthropod-borne viral disease in humans [1]. Approximately 2.5 billion people live in regions at risk and 50-100 million infections occur annually, resulting in half a million individuals suffering from dengue hemorrhagic fever (DHF) and more than 25,000 deaths [2]. The total yearly cost of treatment can reach US $2 billion, based on figures from five countries in the Americas (Brazil, El Salvador, Guatemala, Panama, and Venezuela) and three countries in Asia (Cambodia, Malaysia, and Thailand) [3]. The reported number of dengue cases has increased dramatically in the past five decades, mainly due to growing vector and human population densities [4].

The agent of dengue virus (DENV) is a member of the genus *Flavivirus *family *Flaviviridae*. It is a single-stranded, positive-sense, RNA virus with a genome of about 11 kb, which can be antigenically divided into four serotypes (DENV-1, DENV-2, DENV-3, and DENV-4). All four serotypes commonly cause a mild febrile illness which may progress to DHF and dengue shock syndrome (DSS) [5].

In the past 10 years, DENV-4 has been re-emerging or emerging in different countries and regions. After an absence of 25 years, DENV-4 genotype II re-emerged in Brazil in 2007 [6], followed by genotype I in northern Brazil in 2010 [7]. The detection of DENV-4 was reported for the first time in Easter Island in 2009, which was serotyped as genotype II [8]. Although rarely reported after the 1970s in India, DENV-4 re-emerged in 2007 and was grouped as genotype I, the same genotype detected in 2009 [9]. However, genotypes I, II, and III of DENV-4 have been detected yearly in Thailand since 1963 [10]. DENV-4 genotype II has been isolated in the Caribbean following introduction from Asia in 1981 [11]. In addition, DENV-4 had a higher rate of dispersion than DENV-2 in the Americas during the study period [12] and the evolutionary rate may increase following geographical expansion [13,14]. DENV-4 replaced DENV-3 as the main epidemic strain in Peru in 2008 [15] and took over DENV-1 as the primary epidemic strain in the Pacific region from 2007 to 2009 [16]. Furthermore, severe disease risk was positively correlated to DENV-4, especially for children after secondary infection following antibody-dependent enhancement (ADE). In Thailand, DENV-4 accounted for 10% of DHF cases in children, and most DENV-4 DHF cases were associated with secondary dengue viral infection [10].

In recent years, much attention has focused on genetic diversity in viral evolution. Viral genetic changes play a critical role in tracing the origin of infection, particularly through sequencing and phylogenetic analyses of complete envelope gene sequences from isolates [5], which may result in more virulent or epidemic strains. The strains from individual serotypes often fall into well-defined genotypes, supported by high bootstrap values restricted to particular geographic regions, which are reflective of the extensive migration of both hosts and vectors [10]. To date, four major genotypes of DENV-4 have been described [17]: genotype I, representing strains from Thailand, the Philippines, Sri Lanka and Japan; genotype II, representing strains from Indonesia, Malaysia, Tahiti, the Caribbean and the Americas; genotype III, representing Thai strains that are distinct from other Thai isolates; and genotype IV, representing sylvatic strains from Malaysia.

Guangzhou, a megalopolis in China, is a representative city that has experienced annual DENV transmission, accounting for more than 50% of the DENV cases in mainland China. In recent years, serotypes DENV-1, DENV-2, DENV-3, and DENV-4 have been sequentially circulated in Guangzhou. The DENV-3 serotype was identified in 2009 and was later replaced by DENV-4 in 2010. The first DENV-4 outbreak in Guangzhou occurred in 1978, which was transmitted from Foshan City, with a second DENV-4 outbreak occurring in 1990 [18,19]. After an absence of 20 years, the third DENV-4 outbreak took place in 2010.

In this paper, we investigated the DENV-4 outbreak in the Jingtai Street community which covered 11.4 square kilometres with a population of 32,567 in 2010. There was a high density of *Aedes albopictus *which was identified as the predominant vector for dengue in Guangzhou, with an average Breteau index (BI) of 8.67 in the first three days after the first autochthonous case was distinguished on September 13, 2010. The survey enabled analysis of the epidemiological distribution, laboratory testing and phylogenetic analyses of envelope gene sequences, using 2 sequences extracted from isolates and 32 sequences published in GenBank.

## Case presentations

### Methods for survey and laboratory analysis

All patients were identified from passive surveillance when seeking medical services or recognized by active searches that were reported to the Notifiable Infectious Disease Report System (NIDRS) within 24 hours after diagnosis, and were followed by a face-to-face interview conducted by the Center for Disease Control and Prevention (CDC). Case definition, serological tests, and viral isolation and identification from sera samples conformed to the Diagnostic Criteria for Dengue Fever (WS216-2008) enacted by the Chinese Ministry of Health [20]. In addition, initial serum specimens of patients and 200 healthy persons for population serosurvey were obtained with ethical approval from the Ethics Committee of the Guangzhou Center for Disease Control and Prevention. Written informed consent was obtained from all patients upon seeking medical service.

A Dengue Duo IgM and IgG Capture ELISA Kit (PanBio, Windsor, Australia) was used to serologically diagnose infections [21]. Viral RNA was extracted using the QLAamp Viral RNA Mini Kit (Qiagen, Hilden, Germany). Three pairs of primers were designed with Primer Express Software Version 3.0 (Applied Biosystems, Foster City, CA, USA, see Additional file 1: Table S1 for details) to amplify the entire DENV-4 envelope gene sequence. RNA samples were used for one-step RT-PCR (TaKaRa, Shiga, Japan). The PCR products were purified using QIA-quick PCR Purification Kits (Qiagen) [22]. The envelope genes were purified and completely sequenced using the BigDye Terminator Cycle Sequencing Kit (Applied Biosystems) applying previously described primers. Sequences assemblies were completed using the SeqMan II software (DNASTAR, Inc., Madison, WI, USA).

The phylogenetic tree was constructed by the neighbour-joining method with a Kimura 2 parameter model using MEGA 4.0 software (http://www.megasoftware.net/mega4/mega.html). One thousand bootstrap repetitions were used for confirmation of the statistical significance of the phylogenetic analysis [22]. The DENV isolates (see Additional file 2: Table S2 for details) used in this analysis were published in GenBank. These isolates represented a wide range of geographic localities, including earlier sampled viruses and two sylvatic strains isolated from monkeys in Malaysia.

### Results

One imported case was confirmed. A total of 18 autochthonous cases were identified that met the case definition, including 7 clinically diagnosed cases and 11 confirmed cases. Of these 18 cases, 16 patients were hospitalized. Sixteen cases were identified from passive reports and 2 cases among 18,324 residents from active searches, which produced an incidence rate of 5.53 per 10,000 people. Moreover, 1 (0.50%) serum sample from a healthy person tested positive for IgM in the serosurvey.

#### Imported case

The index imported patient, a 26-year-old female Guangzhou resident from the Yuexiu District, had been on vacation in Bangkok from August 25 to September 1, 2010. On the evening of August 31, prior to her flight back to Guangzhou, she suffered from a sudden fever with temperature of 38.5°C, followed by cephalgia, malaise, arthralgia and exanthema on the forearms and legs. The patient fully recovered 7 days after onset with no hemorrhagic manifestation or other severe alternations. Before her tour to Thailand, she was healthy and reported no febrile illness. After her return, on September 2, she visited a friend living on a fifth floor apartment in Jingtai Street community. On the basis of the patient's symptoms and dengue prevalence in Bangkok, dengue fever (DF) was confirmed and virus isolate D10168-GZ (GenBank accession no. JN029829, submitted by the Guangdong Center for Disease Control and Prevention, China) was found in her serum. Interestingly, IgM and IgG were negative on September 4, but were present on September 8, 2010.

#### Secondary autochthonous cases

**Figure 1:**
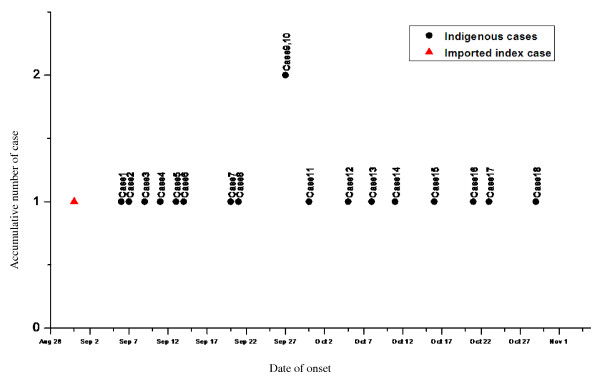
**The dynamic of spread from the imported index case to 18 autochthonous cases in the DENV-4 genotype II outbreak from Jingtai Street in Guangzhou, China, in 2010**.

All 18 autochthonous cases were confined to the Jingtai Street community in Guangzhou. The onset dates of these cases ranged from September 6 to October 29, 2010, without a significant peak occurring on any single day. Eleven cases (61.11%) were reported in September: two on September 27 and one each on September 6, 7, 9, 11, 13, 14, 20, 21 and 30. Seven cases (38.89%) occurred in October: one each on October 5, 8, 11, 16, 21, 23 and 29 (see Figure 1 for the dynamic of spread). The epidemic lasted 53 days, with a median interval from time of onset to report of six days (range: 2-12 days). The first autochthonous case developed illness on September 6. She lived on the third floor in the same building of the friend visited by the imported index case on September 2. The other autochthonous cases had no obvious gathering activities but all lived in the Jingtai Street community. The time distribution indicated a continuous dissemination, which was confined to a specific period.

The infected individuals consisted of eight males and 10 females with sex ratio of 1:1.25 and an average age of 41.68 years (range: 16-82 years). The ages included two people aged 10-19 years, five aged 20-29 years, two aged 30-39 years, one aged 40-49 years, four aged 50-59 years, two aged 60-69 years and two that were more than 70 years old.

The most commonly reported clinical manifestations are listed in Table 1. All patients had fever (100%), 13 cases (72.22%) reported skin petechiae, 10 cases (55.56%) experienced rashes, eight (44.44%) suffered from myalgia and three (16.67%) from arthralgia. The other reported symptoms included malaise (88.89%), cephalalgia (72.22%), facial flushing (11.11%), conjunctival congestion (22.22%), orbital pain (11.11%) and vomiting (5.56%). Clinical blood tests indicated leukocytopenia in 16 cases (88.89%) and thrombocytopenia in 12 cases (66.67%). The median duration of illness was 7 days (range: 3-14 days).

**Table 1:** Symptoms observed in the 18 autochthonous dengue cases from Jingtai Street in Guangzhou, 2010

Symptoms	No. of cases
Fever	18 (100%)
Cephalalgia	13 (72.22%)
Arthralgia	3 (16.67%)
Myalgia	8 (44.44%)
Malaise	16 (88.89%)
Rash	10 (55.56%)
Facial flushing	2 (11.11%)
Skin petechiae	13 (72.22%)
Conjunctival congestion	4 (22.22%)
Orbital pain	2 (11.11%)
Vomiting	1 (5.56%)
Leukocytopenia	16 (88.89%)
Thrombocytopenia	12 (66.67%)

Indigenous cases are listed in Table 2. A total of 15 blood specimens were submitted with a median time interval from onset to sampling of six days (range: 1-12 days). Fourteen serum samples tested positive for IgM against DENV and seven for IgG against DENV. Five DENV-4 strains were isolated with an isolate rate of 33.33% and an interval median of six days (range: 3-7 days) from sampling date to onset of symptoms.

**Table 2:** The sex, age, antibody and viral isolation profiles of the 18 indigenous cases from the DENV-4 outbreak from Jingtai Street, Guangzhou, China, 2010

**Num**.	Age(yr)/Gender	Date of Sampling	IgM	IgG	Viral isolation
Case 1	30/F	9/13/2010	+	+	--
Case 2	31/F	9/13/2010	+	-	DENV-4
Case 3	29/F	ND	ND	ND	ND
Case 4	55/M	9/18/2010	+	+	DENV-4
Case 5	56/M	9/21/2010	+	+	--
Case 6	63/M	9/26/2010	+	+	--
Case 7	61/F	9/23/2010	+	-	DENV-4
Case 8	19/F	9/27/2010	+	-	DENV-4
Case 9	16/M	9/29/2010	+	-	DENV-4
Case 10	82/M	9/28/2010	-	-	--
Case 11	80/M	10/06/2010	+	+	--
Case 12	25/M	ND	ND	ND	ND
Case 13	25/F	10/11/2010	+	-	--
Case 14	22/F	ND	ND	ND	ND
Case 15	40/F	10/22/2010	+	-	--
Case 16	27/M	10/27/2010	+	+	--
Case 17	54/F	10/29/2010	+	+	--
Case 18	54/F	11/04/2010	+	-	--

Note: Num 3, Num 12 and Num 14 were clinically diagnosed cases and no serum was sampled. ND: not done. --:no virus was isolated. IgM and IgG results were from the first serums collected once patients sought medical service.

#### Phylogenetic analysis

Envelope genes of five isolates from the autochthonous cases and one isolate from the index imported case were 1485nt in length, with a homology of 99.9-100%. The first isolate of the autochthonous cases was the isolate Guangzhou 10660 (GenBank accession no. JN599977). Guangzhou 10660 shared 100% homology with D10168-GZ that was isolated in China in 2010 and imported from Thailand, and also shared 98.9% homology with the 02-12-1HuNIID strain isolated in 2002 in Japan which also was imported from Thailand (see Additional file 3: Table S3 for homology details).

**Figure 2:**
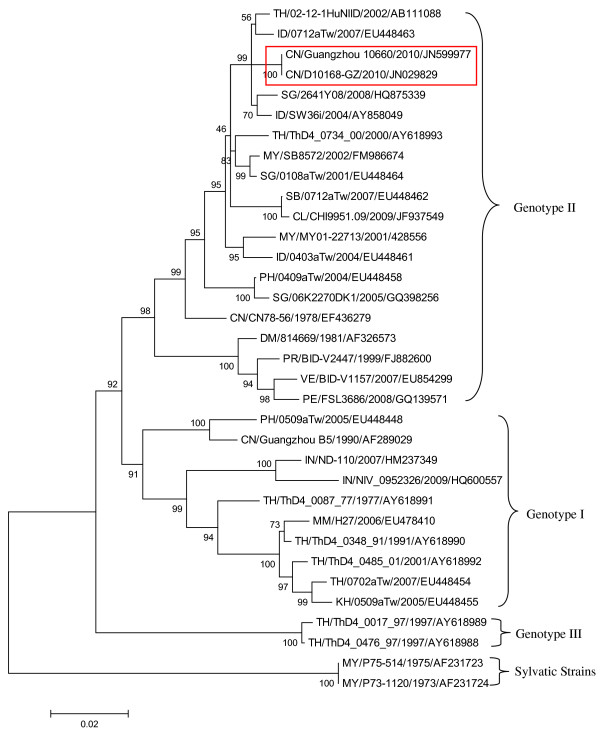
**The phylogenetic analysis of the envelope gene sequences of DENV-4**. The phylogenetic tree was constructed by the neighbour-joining method with a Kimura 2 parameter model using MEGA 4.0 software. Bootstrap values were set for 1000 repetitions and were placed over each main node of the tree. Name rule: country/strain name/isolated year/GenBank accession number. CL: Chile; CN: China; DM: Dominica; ID: Indonesia; IN: India; KH: Cambodia; MM: Myanmar; MY: Malaysia; PE: Peru; PH: the Philippines; PR: Puerto Rico; SB: Solomon; SG: Singapore; TH: Thailand; VE: Venezuela.

Phylogenetically, as demonstrated in Figure 2, Guangzhou 10660 and D10168-GZ are identical, and are located in the same clade and grouped into DENV-4 genotype II. These strains are closely related to 02-12-1HuNIID, and are clustered within the same minor clade as 0712TW isolated in Taiwan in 2007 and imported from Indonesia, SW36i isolated in Indonesia in 2004, and 2641Y08 isolated in Singapore in 2008. These strains are distantly related to strain CN78-56 which was grouped into the genotype II clade which was isolated in China in 1978, and remarkably remote from the Guangzhou B5 strain which isolated in China in 1990 and was grouped to the genotype I clade.

## Conclusions

**Figure 3:**
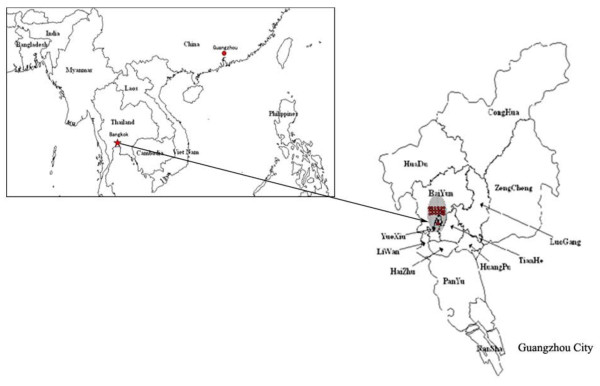
**The source of the DENV-4 genotype II outbreak in Guangzhou City, 2010**. The map in the box shows Southeast Asia. The right map is an enlargement of Guangzhou City, where "▲" denotes the imported index case, a Guangzhou resident who travelled back from Thailand, and "●" represents the 18 secondary autochthonous cases initiated by the imported index case.

DENV-4 has not been identified in local outbreaks in mainland China since the 1990 DENV-4 genotype I outbreak. Based on epidemiological and phylogenetic analyses, this current outbreak was confirmed as DENV-4 genotype II and initially traced to the imported index case, a Guangzhou resident who travelled back from Thailand. The DENV-4 genotype II spread rapidly (Figure 3), but was not classified as a re-emergence of the DENV-4 outbreaks from 1990 and 1978.

Dengue viruses are serologically classified into four antigenically distinct serotypes (DENV-1, DENV-2, DENV-3 and DENV-4). Different genotypes were included in the same serotype according to envelope gene sequences and confined to specific geographical areas [23]. The envelope gene encodes an envelope protein, which induces membrane fusion, and confers protective immune responses by eliciting neutralizing, antifusion and replication-enhancing antibodies [17]. The envelope gene is the most commonly gene surveyed in dengue molecular epidemiological studies [24]. Though the cases were divergent, based on sampling time and patient addresses, the DENV-4 isolates were determined to be from the same origin.

Phylogenetics significantly contributed to the understanding of the relationship among the strains from different areas and times, and to determining whether the epidemic had re-emerged or was newly introduced. The results indicated that the strain responsible for the outbreak in Guangzhou was introduced from Thailand, where DENV-4 had circulated for a long time. This strain was not a re-emergence of the DENV-4 outbreak of 1990. Furthermore, results suggested that strain isolated from Guangzhou in 2010 had evolved from the 2002 Thailand strains.

In China, dengue is thought to be an imported epidemic disease [25]. In the past decades, severe dengue epidemics have occurred in Southeast Asian countries, including Thailand, Singapore, Indonesia, and the Philippines, from which dengue was exported to adjacent countries [26]. Moreover, this community outbreak also was traced to Thailand, which is a major reservoir and epicentre for dengue virus [10].

Guangzhou is the capital city of the Guangdong Province in southern China, located at 112°57'E to 114°3'E and 22°26'N to 23°56'N. Guangzhou has a current population of more than 10 million and a humid subtropical climate influenced by the Asian monsoon [27]. In addition, Guangzhou is one of the most important commercial centers in China and Southeast Asia, and has a large demographic exchange coupled with business, tourism and labour service. Guangzhou is a hub for a large number of tourists and is therefore connected to large and busy roadways, railways, and airway systems that allow easy access to the most populous part of China. Furthermore, the local *Aedes albopictus *populations are large and susceptible to dengue virus. As a result, the city is known as the most important point for the introduction and dissemination of DENV in China. Indeed, Guangzhou has accounted for more than 50% of the dengue cases in mainland China since 1978. The recent introduction of DENV-4 in this state should be of a great concern because of its potential to spread to the rest of China.

The imported index case, a Guangzhou resident who travelled back from Thailand, spread the virus from the Yuexiu District in Guangzhou. This case did not arouse attention until the end of the outbreak. Therefore, early detection of cases and a rapid public health response might prevent the import of a dengue virus which may then lead to an outbreak [28]. As Guangzhou is an ideal location for a dengue epidemic, DENV-4 is a serious threat. Thus, the Bureau of Health should prepare a plan to adopt control measures to avoid or minimize spread of this serotype, provide clinical management, organize healthcare, and maintain an efficient surveillance system to reduce the threat of dengue outbreaks [29].

## Consent

Written informed consent was obtained from the patients for publication of this case report.

## Abbreviations

DENV: Dengue virus; DF: Dengue fever; DHF: Dengue hemorrhagic fever; DSS: Dengue shock syndrome; ADE: Antibody-dependent enhancement; CDC: Center for Disease Control and Prevention; ELISA: Enzyme-linked immunosorbent assay; NIDRS: The Notifiable Infectious Disease Report System; BI: Breteau index.

## Competing interests

The authors declare that they have no competing interests.

## Authors' contributions

J-QL, Y-ZC, LL, X-XC, L-JH conceived of and designed this study and drafted the manuscript. J-QL, Y-ZC, LL, X-XC collected the data and performed the statistical and phylogenetic analyses. J-QL, Y-ZC, HP conducted the laboratory tests. DB, F-CX, WM made significant contributions by providing assistance in the data analysis and laboratory tests. All authors have read and approved the final manuscript.

## Pre-publication history

The pre-publication history for this paper can be accessed here:

http://www.biomedcentral.com/1471-2334/12/87/prepub

## Supplementary Material

Additional file 1 **Table S1 The three pairs of primers used to amplify and sequence the entire DENV-4 envelope gene**. Click here for file

Additional file 2 **Table S2 The DENV-4 reference strains for phylogenetic analysis**. Click here for file

Additional file 3 **Table S3 The homologies among the isolates and different strains from GenBank**. Click here for file
