# Supplementary material for: Emergence of dengue virus 4 genotype II in Guangzhou, China, 2010: Survey and molecular epidemiology of one community outbreak
Source: BMC Infect Dis. 2012 Apr 12;12:87. doi: 10.1186/1471-2334-12-87 (PMC3375192; doi:10.1186/1471-2334-12-87)
Supplement: Additional file 1 — Table S1 The three pairs of primers used to amplify and sequence the entire DENV-4 envelope gene. [file 1471-2334-12-87-S1.DOC]

**Additional file 1**

**Table S1. The three pairs of primers used to amplify and sequence the entire DENV-4 envelope gene**

| Primer | Nucleotide sequence（5’～3’） | Genome position | Amplification size（bp） |
| --- | --- | --- | --- |
| E1F | GACAACAGAAGGAATCAATAAGTG | 518～541 | 730 |
| E1R | CGTTGCCCCACCCTCTATCT | 1248～1267 |
| E2F | CATAACCACGGCAACAAGAT | 1139～1158 | 956 |
| E2R | GCACTGTCTCCTACACCTATTACTAT | 2095～2120 |
| E3F | AAAAGTCAAGTATGAGGGTGCT | 1904～1925 | 1003 |
| E3R | TGCCAAATCCATAGTCTTCC | 2907～2926 |
