# Supplementary material for: Emergence of dengue virus 4 genotype II in Guangzhou, China, 2010: Survey and molecular epidemiology of one community outbreak
Source: BMC Infect Dis. 2012 Apr 12;12:87. doi: 10.1186/1471-2334-12-87 (PMC3375192; doi:10.1186/1471-2334-12-87)
Supplement: Additional file 2 — Table S2 The DENV-4 reference strains for phylogenetic analysis. [file 1471-2334-12-87-S2.DOC]

**Additional file 2**

**Table S2.** The DENV-4 reference strains for phylogenetic analysis

| No. | Virus strain | Isolation year | Country of origin | Sequence | GenBank Accesion # |
| --- | --- | --- | --- | --- | --- |
| 1 | ThD4_0087_77 | 1977 | Thailand | genome | AY618991 |
| 2 | ThD4_0348_91 | 1991 | Thailand | genome | AY618990 |
| 3 | ThD4_0017_97 | 1997 | Thailand | genome | AY618989 |
| 4 | ThD4_0476_97 | 1997 | Thailand | genome | AY618988 |
| 5 | ThD4_0734_00 | 2000 | Thailand | genome | AY618993 |
| 6 | ThD4_0485_01 | 2001 | Thailand | genome | AY618992 |
| 7 | Guangzhou B5 | 1990 | China | genome | AF289029 |
| 8 | 814669 | 1981 | Dominica | genome | AF326573 |
| 9 | 02-12-1HuNIID | 2002 | Thailand | envelope | AB111088 |
| 10 | SW36i | 2004 | Indonesia | envelope | AY858049 |
| 11 | 0712aTw | 2007 | Indonesia | envelope | EU448463 |
| 12 | 0108aTw | 2001 | Singapore | envelope | EU448464 |
| 13 | CN78-56 | 1978 | China | envelope | EF436279 |
| 14 | CHI9951.09 | 2009 | Chile | envelope | JF937549 |
| 15 | FSL3686 | 2008 | Peru | envelope | GQ139571 |
| 16 | BID-V2447 | 1999 | Puerto Rico | genome | FJ882600 |
| 17 | NIV_0952326 | 2009 | India | envelope | HQ600557 |
| 18 | ND-110 | 2007 | India | envelope | HM237349 |
| 19 | 2641Y08 | 2008 | Singapore | envelope | HQ875339 |
| 20 | SB8572 | 2002 | Malaysia | envelope | FM986674 |
| 21 | 0712aTw | 2007 | Solomon | envelope | EU448462 |
| 22 | 0403aTw | 2004 | Indonesia | envelope | EU448461 |
| 23 | MY01-22713 | 2001 | Malaysia | envelope | AJ428556 |
| 24 | BID-V1157 | 2007 | Venezuela | genome | EU854299 |
| 25 | 0409aTw | 2004 | Philippines | envelope | EU448458 |
| 26 | 0509aTw | 2005 | Philippines | envelope | EU448448 |
| 27 | 06K2270DK1 | 2005 | Singapore | genome | GQ398256 |
| 28 | 0509aTw | 2005 | Cambodia | envelope | EU448455 |
| 29 | H27 | 2006 | Myanmar | envelope | EU478410 |
| 30 | 0702aTw | 2007 | Thailand | envelope | EU448454 |
| 31 | P73-1120 | 1973 | Malaysia | envelope | AF231724 |
| 32 | P75-514 | 1975 | Malaysia | envelope | AF231723 |
