# Supplementary material for: Emergence of dengue virus 4 genotype II in Guangzhou, China, 2010: Survey and molecular epidemiology of one community outbreak
Source: BMC Infect Dis. 2012 Apr 12;12:87. doi: 10.1186/1471-2334-12-87 (PMC3375192; doi:10.1186/1471-2334-12-87)
Supplement: Additional file 3 — Table S3 The homologies among the isolates and different strains from GenBank. [file 1471-2334-12-87-S3.DOC]

**Additional file 3**

**Table S3.** The homologies among the isolates and different strains from GenBank

| NUM | Strains | 1 | 2 | 3 | 4 | 5 | 6 | 7 | 8 | 9 | 10 | 11 | 12 |
| --- | --- | --- | --- | --- | --- | --- | --- | --- | --- | --- | --- | --- | --- |
| 1 | Guangzhou 11290 |  | 100.0 | 99.9 | 99.9 | 100.0 | 100.0 | 98.9 | 98.6 | 96.7 | 93.9 | 98.8 | 98.5 |
| **2** | **Guangzhou 10660** |  |  | **99.9** | **99.9** | **100.0** | **100.0** | **98.9** | **98.6** | **96.7** | **93.9** | **98.8** | **98.5** |
| 3 | Guangzhou 10579 |  |  |  | 99.9 | 99.9 | 99.9 | 98.8 | 98.5 | 96.6 | 93.8 | 98.7 | 98.4 |
| 4 | Guangzhou 11870 |  |  |  |  | 99.9 | 99.9 | 98.8 | 98.5 | 96.6 | 93.8 | 98.7 | 98.4 |
| 5 | Guangzhou 10931 |  |  |  |  |  | 100.0 | 98.9 | 98.6 | 96.7 | 93.9 | 98.8 | 98.5 |
| 6 | D10168-GZ |  |  |  |  |  |  | 98.9 | 98.6 | 96.7 | 93.9 | 98.8 | 98.5 |
| 7 | 02-12-1HuNIID |  |  |  |  |  |  |  | 99.2 | 97.0 | 93.9 | 99.3 | 99.1 |
| 8 | 0712aTw |  |  |  |  |  |  |  |  | 96.9 | 94.3 | 99.0 | 98.7 |
| 9 | CN78-56 |  |  |  |  |  |  |  |  |  | 94.9 | 97.1 | 96.6 |
| 10 | Guangzhou B5 |  |  |  |  |  |  |  |  |  |  | 87.1 | 88.1 |
| 11 | SW36i |  |  |  |  |  |  |  |  |  |  |  | 95.3 |
| 12 | 2641Y08 |  |  |  |  |  |  |  |  |  |  |  |  |
